# Diagnosis of *Giardia* spp. in ruminants at Southern Brazil

**DOI:** 10.29374/2527-2179.bjvm006524

**Published:** 2024-12-18

**Authors:** Tamires Silva dos Santos, Giulia Ribeiro Meireles, Camila Gonçalves da Silveira, Gabrielle Torres Cotta de Mello, Stanrley Victor Nascimento da Silva, Julia Somavilla Lignon, Natália Soares Martins, Diego Moscarelli Pinto, Felipe Geraldo Pappen

**Affiliations:** 1 Veterinarian, Laboratório do Grupo de Estudos em Enfermidades Parasitárias, Departamento de Veterinária Preventiva, Universidade Federal de Pelotas, Pelotas, Rio Grande do Sul, Brazil.

**Keywords:** cattle, giardiasis, public health, protozoa, sheep, bovinos, giardíase, saúde pública, protozoários, ovinos

## Abstract

*Giardia* spp. is a flagellated protozoan that parasitizes the small intestine of various mammals, birds, and amphibians, being transmitted via the fecal-oral route. Giardiasis is one of the most prevalent parasitic diseases in both developed and developing countries, with a prevalence of 0,1-5% and 20-30%, respectively, and is correlated with poor hygiene practices, such as irregular handwashing, which consequently contaminates food when handled. Cattle and sheep are sources of infection for humans due to the zoonotic potential of the species that affect them, especially calves, which play an important role in the dissemination of the parasite in the environment by excreting 10^6^ cysts per gram of feces, contaminating water sources, which, even when treated, only reduce and do not eliminate the protozoan. This study investigated the prevalence of *Giardia* spp. in ruminants in the southern region of Rio Grande do Sul, Brazil. Between June 2023 and April 2024, 384 fecal samples from young cattle and sheep were analyzed, collected directly from the rectal ampulla and subjected to coprological tests at the Laboratory, used the zinc sulfate centrifugal flotation technique to visualize protozoan cysts and calculate their prevalence. The results showed that 19,15% of sheep (27/141), 13,99% of cattle (34/243) and 15.88% in both species (61/384) tested positive for *Giardia* spp. This study revealed a significant prevalence of *Giardia* spp. in young ruminants in the southern region of Rio Grande do Sul, posing an important zoonotic risk.

## Resumo

Giardia spp. é um protozoário flagelado que parasita o intestino delgado de vários mamíferos, aves e anfíbios, sendo transmitido pela via fecal-oral. A giardíase é uma das doenças parasitárias mais prevalentes tanto em países desenvolvidos quanto em desenvolvimento, com prevalência de 0,1-5% e 20-30%, respectivamente, e está correlacionada com práticas precárias de higiene, como lavagem irregular das mãos, que consequentemente contamina os alimentos quando manipulados. Bovinos e ovinos são fontes de infecção para humanos devido ao potencial zoonótico das espécies que os acometem, especialmente bezerros, que desempenham importante papel na disseminação do parasita no ambiente ao excretar 10^6^ cistos por grama de fezes, contaminando fontes de água, que, mesmo tratadas, apenas reduzem e não eliminam o protozoário. Este estudo investigou a prevalência de *Giardia* spp. em ruminantes na região sul do Rio Grande do Sul, Brasil. Entre junho de 2023 e abril de 2024, foram analisadas 384 amostras fecais de bovinos e ovinos jovens, coletadas diretamente da ampola retal e submetidas a testes coprológicos no Laboratório, utilizando a técnica de centrífuga-flotação com sulfato de zinco para visualização de cistos de protozoários e cálculo de sua prevalência. Os resultados mostraram que 19,15% dos ovinos (27/141), 13,99% dos bovinos (34/243) e 15,88% em ambas as espécies (61/384) apresentaram resultados positivos para *Giardia* spp. Este estudo revelou uma prevalência significativa de Giardia spp. em ruminantes jovens na região sul do Rio Grande do Sul, representando um importante risco zoonótico.

## Introduction

*Giardia* spp. is a flagellated protozoan that parasitizes the small intestine of mammals, birds, reptiles and amphibians worldwide (Ryan & Zahedi, 2019). There are numerous species of *Giardia* spp. that infect various hosts, and transmission occurs via the fecal-oral route, either through direct contact with infected humans or animals and indirectly through the ingestion of food and water contaminated with cysts (Ryan & Zahedi, 2019; Santin, 2020).

Although it is widely associated with infections in companion animals (e.g., dogs and cats) and the risk of transmission to humans, its incidence in ruminants is also significant, requiring detailed investigations in the context of public health (Mateusa et al., 2023; Squire & Ryan, 2017). Cattle, sheep, and goats are often affected by different species of *Giardia* spp. (e.g., *Giardia duodenalis* and *Giardia bovis*), contributing to the spread of the parasite in the environment and increasing the likelihood of transmission to humans (Mateusa et al., 2023; Squire & Ryan, 2017).

The prevalence of *Giardia* spp. in livestock is high, with rates in cattle reaching up to 74% (Moreira et al., 2020). Thus, *G. duodenalis* (syn. *G. intestinalis, G. lamblia)* is subdivided into eight assemblages: A and B, which are responsible for infecting humans and other domestic and wild mammals; C and D – canines; E – cattle and other hoofed animals; F – domestic cats; G – rats; and H – pinnipeds (Moreira et al., 2020; Ryan & Cacciò, 2013; Ryan & Zahedi, 2019; Taghipour et al., 2022).

In cattle and sheep, the disease can be transmitted by different groups, with group E being the most commonly described, as well as assemblages A, B, and D, which have also been identified in these animals. Assemblages A and B are characterized by a high zoonotic potential due to their wide range of hosts. Additionally, studies report the occurrence of group E in humans in several countries, including Brazil (Fantinatti et al., 2016; Zhao et al., 2024).

Affected animals are usually asymptomatic but may exhibit clinical signs such as diarrhea, lethargy, weight loss, and consequently, a decrease in production (meat, milk, and wool) (Sá et al., 2020). On the other hand, in countries like Brazil, giardiasis affects more than 50% of the population, with children being the most impacted, especially due to the habit of not washing their hands frequently and having direct contact with infected domestic animals. In the chronic phase of the disease, the protozoan can cause intestinal absorption problems, which can lead to weight loss, iron deficiency and anemia (Fakhri et al., 2021; Monteiro, 2017; Sá et al., 2020).

Considering the importance of Rio Grande do Sul in cattle and sheep farming, along with the economic losses due to parasitic diseases that affect these animals and the impact of giardiasis on public health, the present study aims to evaluate the prevalence of *Giardia* spp. in ruminants from farms located in the southern region of Rio Grande do Sul, Brazil.

## Materials and methods

### Data collection for the study

To conduct the study, fecal samples from ruminants (cattle and sheep) received and processed in the laboratory of the Parasitic Diseases Study Group of the Federal University of Pelotas during the period from June 2023 to April 2024 were used. These samples came from 63 farms in the southern region of Rio Grande do Sul, Brazil.

A total of 384 samples were analyzed: 141 from sheep and 243 from young cattle, up to one year of age. These samples were collected directly from the rectal ampoule using a plastic bag or sterile glove, identified, placed in refrigerated isothermal containers and transported to the laboratory by the responsible veterinarian, where they were subjected to coprological tests to diagnose intestinal protozoa.

### Laboratory examination

To visualize *Giardia* spp. cysts, the centrifuge-flotation technique in zinc sulfate described by Faust et al. (1938), modified by Monteiro (2017), was used. This method allows for the identification of helminth eggs, protozoan cysts, or oocysts. The technique is considered a qualitative diagnostic method based on the principle of the flotation of eggs, cysts, or oocysts toward the coverslip.

The method involves weighing 2 grams of feces, macerating and homogenizing it with 15 ml of 33% zinc sulfate (ZnSO4) solution, and then performing sieving. After this process, the solution is placed into a Falcon® tube until a meniscus forms. A coverslip is then placed on top, and the tube is transferred to a centrifuge and spun for 5 minutes at 1,500 to 2,000 revolutions per minute. After centrifugation, the coverslip is removed, and a drop of Lugol's iodine solution is added. The coverslip is then placed on a microscope slide and examined under optical microscopy (10x to 40x magnification). After processing the samples, the presence or absence of *Giardia* spp. cysts was observed. The cysts, measuring approximately 12μm, are characterized by having an ovoid shape and the presence of four nuclei, and are identified as described by Monteiro (2017).

### Statistical analysis

The prevalence was calculated as the proportion of positive cases relative to the total number of individuals examined, expressed as a percentage (Meek & Willeberg, 1987; Rothman, 2012). The formula used for the calculation is:

Prevalence (%) = (Number of positive cases ÷ Total number of individuals) × 100

### Results

**Figure 1:**
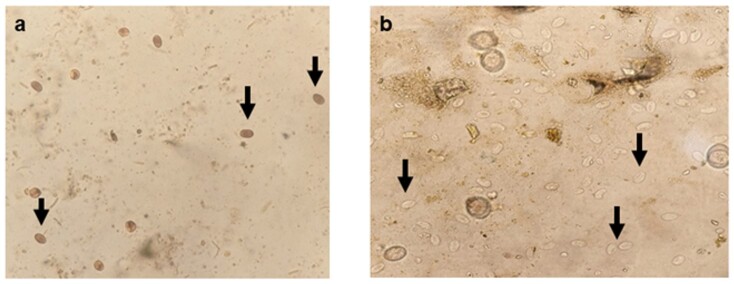
Optical microscopy images of Giardia spp. cysts at 400X magnification. (a) Giardia spp. cysts stained with Lugol in cattle; (b) Giardia spp. cysts in sheep. The black arrow indicates Giardia spp. cysts.

In this study, the prevalence of *Giardia* spp. was assessed in two species of ruminants. Of the 141 sheep analyzed, 27 tested positive, corresponding to a prevalence of 19.15%. Among the 243 cattle tested, 34 were positive, resulting in a prevalence of 13.99%. Therefore, considering both species, the overall prevalence observed was 15.88%. Figure 1 presents images of the protozoan cysts, recorded for visual analysis.

## Discussion

The prevalence results found were of 13,99% (34/233) of cattle and 19,15% (27/141) of sheep positive for *Giardia* spp., which differ from the data found in other studies such as Oliveira et al. (2020), who analyzed 100 sheep samples in Brazil, finding 33% of animals positive for *Giardia* spp., a number higher than observed in this study. Regarding cattle, Toledo et al. (2017) found a lower number, 7,6% positive from 937 fecal samples of calves. Both authors used microscopy as a diagnostic technique, although with different methods.

Studies using molecular techniques, such as Zhao et al. (2024), found a higher number of positive animals, with 24% of 749 cattle samples positive, and Paz e Silva et al. (2014) found a prevalence of 34% positive using polymerase chain reaction (PCR) and 37% using enzyme-linked immunosorbent assay (ELISA) from 100 sheep fecal samples. According to a study conducted on 500 sheep fecal samples, there was a significant difference in results depending on the diagnostic method chosen: while microscopy showed a prevalence of 8,4%, PCR indicated 10,2%, demonstrating that the latter has higher sensitivity and specificity (Çelik et al., 2023; Santin, 2020).

Although molecular methods are more reliable compared to coproparasitological diagnosis (Santin, 2020), the latter is a lower-cost, faster, and more accessible alternative for most producers, especially for large herds.

Geurden et al. (2010) suggest that due to the intermittent shedding of *Giardia* spp. cysts, the diagnosis of the protozoan should be based on multiple samples, especially during the chronic phase. Paz e Silva et al. (2012) mention that it would be possible to estimate the prevalence of giardiasis at 100% by analyzing more than one sample from the same animal over a period of about three days. In this context, the results found in this study may be underestimated, as only one sample per animal was analyzed.

The diagnostic methods for *Giardia* spp. are optical microscopy, antigen detection, or PCR (Geurden et al., 2010; Santin, 2020). Through microscopic examination, it is possible to observe the protozoan trophozoites and cysts using both direct and indirect techniques after concentration with sucrose, zinc sulfate, or formalin. This method has advantages such as speed and only the cost of the materials used, but disadvantages include the need for a trained and qualified person for diagnosis and lower sensitivity compared to other methods (Geurden et al., 2010). In this context, the centrifugation flotation technique used in this study is more recommended, as it does not distort *Giardia* spp. cysts, unlike techniques that use supersaturated solutions such as salt and sugar (Monteiro, 2017).

*Giardia* spp. infection in cattle and sheep is associated with various risk factors, including contaminated environments, high population density, the use of untreated water sources, and inadequate management practices (Zhao et al., 2023). The concentration of animals in intensive systems and confined areas facilitates the spread of cysts in the environment (Geurden et al., 2010). Additionally, young animals are more susceptible to infection due to their developing immune systems (Zhao et al., 2024). Stressful conditions, such as weaning and transportation, can also impair the immune response of animals, increasing their vulnerability to giardiasis (Park et al., 2023).

In animal production, giardiasis causes economic losses due to asymptomatic infections and diarrhea (Guimarães et al., 2009; Santin, 2020). In cattle, the disease is related to weight gain; a study observed that the average daily gain in negative calves is higher than in animals positive for *Giardia* spp. (Urie et al., 2018). In sheep, diarrhea affects growth reduction and consequently decreases carcass weight in lambs (Jacobson et al., 2016; Zahedi et al., 2020).

Cattle and sheep are sources of infection for humans due to the zoonotic potential of the species affecting them (Geurden et al., 2010; Ryan & Cacciò, 2013). Calves, in particular, play an important role in the spread of the parasite in the environment by excreting 10^6^ cysts per gram of feces, contaminating water sources, which, even when treated, only reduce rather than eliminate the protozoan (Mateusa et al., 2023; Toledo et al., 2017).

The genotypes of *Giardia* spp. classified as zoonotic mainly include assemblages A and B, which have the ability to infect both humans and animals, such as ruminants (cattle and sheep). Although the samples in this study were not genotyped in this region, it is important to highlight that these assemblages are widely recognized as zoonotic in various epidemiological studies conducted in countries such as China, Korea, and Iraq (Alseady et al., 2023; Park et al., 2023; Zhao et al., 2024). In Brazil, Paz e Silva et al. (2012) identified genotype A in cattle feces in the state of São Paulo. In the region close to the current study, Jeske et al. (2022) investigated immunosuppressed patients treated in a hospital in Pelotas, covering municipalities in southern Rio Grande do Sul, and identified genotypes A, B, C, and D in fecal samples from these patients.

Transmission to other hosts can occur through contaminated water, irrigation of crops (fruits and vegetables) with wastewater containing animal fecal material, slaughterhouse effluents, and direct oro-fecal contact between people and infected animals, especially in the case of veterinarians, farmers, and zoo workers (Onursal & Icgen, 2023; Tawana et al., 2023; Zahedi et al., 2020).

Giardiasis is one of the most common parasitic diseases worldwide, occurring in both developed and developing countries, with a prevalence of 0.1-5% and 20-30%, respectively (Ehsan et al., 2015; Tawana et al., 2023; Zini et al., 2021), and is correlated with poor hygiene practices, such as irregular handwashing, which can consequently contaminate food during handling (Colli et al., 2015).

In southern Rio Grande do Sul, the prevalence of *Giardia* spp. was 66.7% in calves and 25% in children, according to a study conducted in the municipality of Capão do Leão, which evaluated 148 cattle and 22 children from 30 dairy farms (Recuero, 2007). Additionally, Jeske et al. (2022) conducted a study in a hospital serving patients from municipalities in the southern part of the state, observing a positivity rate of 17.3% for *Giardia* spp. among patients. These are the only studies found in the region on the prevalence of *Giardia* spp., highlighting the need for future research to deepen knowledge on the circulation of the protozoan and associated risk factors.

The southern region of the state is considered developed in terms of human development and veterinary services for companion animals, compared to other regions (Coelho et al., 2017). Although most municipalities in this region have basic sanitation and potable water treatment, Zini et al. (2021) detected *Giardia* spp. cysts in water supply stations in Rio Grande do Sul. The state’s economic model, which uses spring areas for water collection, may favor protozoan contamination, highlighting deficiencies in urban sewage treatment and livestock waste management. The circulation of *Giardia* spp. is considered endemic in the region, with agricultural activities and insufficient basic sanitation being identified as major sources of water contamination.

Annually, 200 million people are infected with *Giardia* spp., making the disease of significant public health importance (Jeske et al., 2022), especially as it affects children, the elderly, and immunocompromised individuals who are the most susceptible to infection (Razzolini et al., 2020). In humans, the clinical manifestations of *Giardia* spp. are quite variable, some cases are asymptomatic, while others may present symptoms such as abdominal pain, nausea, vomiting, acute and chronic diarrhea, weight loss, and in severe cases, lead to the death of the patient (Ryan & Cacciò, 2013; Tawana et al., 2023; Yin et al., 2022).

## Conclusion

This study revealed a significant prevalence of *Giardia* spp. in young ruminants in the southern region of Rio Grande do Sul, representing an important zoonotic risk.
